# The role of IL-10 in *Mycobacterium avium* subsp. *paratuberculosis* infection

**DOI:** 10.1186/s12964-016-0152-z

**Published:** 2016-12-01

**Authors:** Tariq Hussain, Syed Zahid Ali Shah, Deming Zhao, Srinand Sreevatsan, Xiangmei Zhou

**Affiliations:** 1 National Animal Transmissible Spongiform Encephalopathy Laboratory and key Laboratory of Animal and Zoonosis of Ministry Agriculture, College of Veterinary Medicine and State key Laboratory of Agrobiotechnology, China Agricultural University, Beijing, 100193 People’s Republic of China; 2 Veterinary Population Medicine Department, College of Veterinary Medicine, University of Minnesota, St Paul, MN USA

**Keywords:** *Mycobacterium avium* subsp. *paratuberculosis* (MAP), Mitogen-activated protein kinase (MAPK), Toll like receptor 2 (TLR2), Interleukin-10 (IL-10), Janus Kinase (JAK), Signal transducer and activators of transcription-3 (STAT3)

## Abstract

*Mycobacterium avium* subsp. *paratuberculosis* (MAP) is an intracellular pathogen and is the causative agent of Johne’s disease of domestic and wild ruminants. Johne’s disease is characterized by chronic granulomatous enteritis leading to substantial economic losses to the livestock sector across the world. MAP persistently survives in phagocytic cells, most commonly in macrophages by disrupting its early antibacterial activity. MAP triggers several signaling pathways after attachment to pathogen recognition receptors (PRRs) of phagocytic cells. MAP adopts a survival strategy to escape the host defence mechanisms via the activation of mitogen-activated protein kinase (MAPK) pathway. The signaling mechanism initiated through toll like receptor 2 (TLR2) activates MAPK-p38 results in up-regulation of interleukin-10 (IL-10), and subsequent repression of inflammatory cytokines. The anti-inflammatory response of IL-10 is mediated through membrane-bound IL-10 receptors, leading to trans-phosphorylation and activation of Janus Kinase (JAK) family receptor-associated tyrosine kinases (TyKs), that promotes the activation of latent transcription factors, signal transducer and activators of transcription 3 (STAT3). IL-10 is an important inhibitory cytokine playing its role in blocking phagosome maturation and apoptosis. In the current review, we describe the importance of IL-10 in early phases of the MAP infection and regulatory mechanisms of the IL-10 dependent pathways in paratuberculosis. We also highlight the strategies to target IL-10, MAPK and STAT3 in other infections caused by intracellular pathogens.

## Background

*Mycobacterium avium* subsp. *paratuberculosis* (MAP) is the causative agent of paratuberculosis or Johne’s disease (JD), chronic progressive granulomatous enteritis in ruminants. This disease was first described by Dr. Heinrich Albert Johne and Dr. Herman Markus in 1895, during examination of a cow’s intestine with a history of reduced milk production and progressive emaciation [1].

The pathogenesis of paratuberculosis is divided into three distinct phases: Silent infection phase, with the appearance of no clinical signs, but animals intermittently shed MAP in the faeces, Subclinical phase: there is an altered immune response with no weight loss or diarrhoea [2], while clinical disease phase: characterized by profuse diarrhoea, marked weight loss, that can be diagnosed by routine MAP detecting procedures [3]. In the clinical phase majority of the animals die due to severe dehydration and emaciation [1, 4]. Apart from common domesticated animals such as cattle, sheep and goats MAP also infect a wide range of other wild ruminants such as deer [5, 6]. The most common route of infection is fecal-oral for all age of animals, while calves can also get the infection via contaminated milk. MAP is also detected in the saliva of cows, indicating that saliva might be a potential mode of transmission [7–9]. Serious economic losses from paratuberculosis occurs due to early culling of the animals with decreased milk production on one hand, but on the other hand loss of capital investments in treating secondary infections, such as mastitis and reduced fertility [10]. Paratuberculosis is also considered a potential zoonotic threat. In 1913, Dalziel reported the link between MAP and human *Morbus Crohn* disease (CD). Several studies have demonstrated the presence of MAP in CD cases, but the causative role of MAP in CD is not established yet [11, 12]. Similarly McNees and colleagues reported that MAP can be cultured from mononuclear cells of Crohn’s disease patient’s but it does not establish the etiology [13].

Immunological responses observed in paratuberculosis begin with a robust T-helper1 (Th1) type response predominantly enhanced interferon-gamma (IFN-γ) production [14, 15]. The hallmark of paratuberculosis is shift in immune response from a cell mediated Th1-type response to a humoral T helper2 (Th2) type response with the progression of disease from a subclinical to clinical phase [16]. Similarly, in ovine model of paratuberculosis, it is observed that the cellular immune response attenuates in natural and experimental MAP infected animals in more advanced phase of disease [17]. MAP rapidly (within 6–24 h) shutdown the pro-inflammatory T cell immune response by promoting immunosuppressive cytokines (IL-10, TGF-β) in in-vitro models . Impairment of CD40 signaling, which is an important macrophage receptor for CD40L on Th1 cells to maintain a Th1 immune response [18]. Mononuclear phagocytic cells (Macrophages), serves as intracellular niche for the MAP survival and multiplication. Macrophages have several pathogen recognition receptors (PRRs), involved in the uptake of mycobacteria [19, 20]. Toll like receptors are family of cell membrane receptors that initiate cell signaling associated with mycobacterium infection [21]. Several studies indicated that pathogenic mycobacteria signal through TLR2 to suppress macrophage antimicrobial responses and antigen presentations [22, 23]. MAP initiates signaling via binding with TLR2 of bovine mononuclear phagocytic cells that result in the activation of MAPK-p38 with a high level of IL-10 production and low level of IL-12 [24–27]. Pathogenic mycobcteria contains mannosylated lipoarabinomannan (Man-LAM) while arabinosylated lipoarabinomannan (Ara-LAM) is usually found in the cell wall of non-pathogenic mycobacteria. Man-LAM is one of the important pathogen associated molecular pattern (PAMP) of MAP that ligate with TLR2 to enhance IL-10 production as well as transient expression of tumor necrosis factor alpha (TNF-α) [26]. Furthermore, it is reported that antigen recognition via TLR2/4 and CD14 in in-vitro and in-vivo lead to oversecretion of IL-10 by disrupting inflammatory responses and generation of adoptive immunity, thereby providing a niche for the persistent survival of pathogen [28].

IL-10 is a potent anti-inflammatory cytokine, crucial for down-regulating pro-inflammatory genes, induced by TLR signaling [29]. IL-10 is not only an essential immunoregulator in host immunity, but it also accounts for the intracellular survival of mycobacterium due to its inhibitory activity against anti-mycobacterial functions of macrophages [30]. The absence of IL-10 leads to better clearance of some pathogens with no enhanced immuno-pathology [31, 32]. The level of IL-10 production depends on the type and strength of the stimulus, while the molecular mechanisms for the regulation of IL-10 differ according to cell type, although some common mechanisms also exist [33]. The main signaling pathways for the production and regulation of IL-10 in phagocytic cells are mitogen-activated protein kinases (MAPKs) pathways, nuclear factor kappa-B (NF-kB) and signal transducer and activators of transcription-3 (STAT3). IL-10 is known to suppress macrophage activation and down-regulate anti-inflammatory and antimicrobial responses such as the production of nitric oxide (NO2), TNF-α, and IL-12 [34, 35]. IL-10 promotes the viability of host cells by inhibiting autophagy, via activating B-cell lypmphoma2 (Bcl2) [36]. Feng and colleagues reported that transgenic mice expressing human IL-10 under the control of major histocompatibility complex class II promoter (hu10Tg) were more susceptible to MAP infection than the wild-type mice. In addition, hu10Tg mice strongly inhibited anti-mycobacterial response of the macrophages such as apoptosis, production of TNF, nitric oxide, and IL-12p40 [37].

Successful phagolysosomal maturation is an important innate immune response for the clearance of intracellular pathogens. MAPK-p38 plays an important role in the signaling cascades during mycobacterial infection [38–40] and is associated with arresting phagosome–lysosome fusion and maturation [41]. MAP interference with phagosome maturation in the infected macrophages depends on the MAPK-p38 signaling pathway activated by Man-LAM and also plays a role in the survival of *Mycobacterium avium* subsp *avium* (MAA) [26]. Interestingly, it was observed that Man-LAM but not Ara-LAM induce IL-10 secretion in LPS stimulating DCs [42]. IL-10 also mediates its anti-inflammatory effects on macrophages by signaling through STAT3 [43, 44]. STAT3 plays an important role as a signaling mediator of IL-6 and IL-10 family members and other cytokines [45]. The blocking of IL-10 in *Mycobacterium tuberculosis* (Mtb) infected macrophages, allows phagosome maturation, but the addition of IL-10 to cells infected with killed Mtb successfully inhibits its maturation, resulting in enhanced survival of Mtb via inhibiting phagosome maturation by IL-10 [46]. Moreover, it is reported that IL-10 secreting transgenic mice were unable to clear the infection and developed bacterial burden after being challenged with BCG (*Mycobacterium bovis*), suggesting that IL-10 help in mycobacterial infection [47]. On the other hand the blocking of IL-10 in Mtb susceptible CBA/J mice minimized the pulmonary bacterial load and improved the animal survival during chronic infection [48]. In MAP infection it has been investigated that the over expression of IL-10 dependent MAPK-p38 [24] is associated with the survival of macrophages by inhibiting the apoptosis phenomenon [35]. IL-10 negatively regulate apoptosis via the activation of STAT3 to promote the transcription of anti-apoptotic and cell cycle progression genes such as BCLXL, CyclinD1, CyclinD2, CyclinD3, and CyclinA, Pim1, c-Myc and p19 (INK4D) [49].

It is clear from previous studies that IL-10 regulation can be a potential hotspot for devising therapeutic measures in intracellular pathogens including MAP. IL-10 regulation can be exploited through recently developed microRNAs (miRNAs) controlling strategies. Several microRNAs regulate the expression of IL-10 by targeting its transcription at 3UTR or by targeting pathways that regulate IL-10 production. Apart from miRNAs, several compounds such as ammonium trichloro (dioxoethylene-O, O’) tellurate (AS101), SD169 and an antihelminth niclosamide can be used to inhibit IL-10 mediated anti-inflammatory activity that has already shown promising results in various infections.

## The role of IL-10 in paratuberculosis

The role of IL-10 is well established in paratuberculosis, as IL-10 plays an important role in the cross talk between innate and adoptive immune responses of the host. IL-10 mediated suppression of IFN-γ secretion in peripheral blood of cattle and goats exposed to MAP infection is also documented [50, 51]. MAP stimulates IL-10 secretion from ovine and bovine monocytes derived antigen presenting cells [52–54]. Coussens and colleagues investigated the expression of cytokine genes in Peripheral blood mononuclear cells (PBMC) of subclinical paratuberculosed and disease-free animals, an increase of IFN-γ gene expression was observed in subclinical animals, while in-vitro stimulation did not show any increase in the expression of IFN-γ. In contrast, IL-10 gene expression was low in unstimulated cells from both groups, but MAP stimulated cells showed an enhanced expression of IL-10 in subclinically infected animals in comparison of number of cytokines, including IFN-γ, TGF-β, TNF-α, IL-1α, IL-4, IL-6, IL-8, and IL-12p35 [55]. Janagama and colleagues investigated that bovine monocyte derived macrophages (BMDM) stimulated with bovine MAP isolate (MAP1018) up-regulated IL-10 at the transcript level and down-regulated TNF-α at both protein and transcript levels in BMDM cells compared with human and sheep MAP (MAP S7565) isolates [56]. Man-LAM of MAP has been identified as one of the ligands that modulates macrophage function and induce a rapid and prolonged expression of IL-10 as well as transient expression of TNF-α [26]. Khalifeh and Stabel also found that IL-10 secretion was elevated upon incubation of live MAP with PBMC from naturally infected cattle, when compared to healthy animals [38]. In ovine paratuberculosis, animals with paucibacillary (type of lesions largely composed of lymphocytes with some macrophages and giant cells and few acid-fast bacteria) disease are more likely to have elevated levels of IFN-γ in illeal tissue while elevated levels of IL-10 are more likely in multibacillary (type of lesions contain few lymphocytes and numerous clusters of macrophages having acid-fast bacteria) animals [57]. Narnaware and colleagues also reported that MAP baison strain upregulate both TGF-β and IL-10 genes in paucibacillary and multibacillary cases in bullocks while a higher magnitude of anti-inflammatory cytokines were observed in animals of multibacillary lesions [58].

IL-10 is an important immunoregulator on one hand, but on the other hand it inhibits anti-mycobacterial activity of macrophages and promotes the intracellular survival of mycobacteria [30]. Previously it was reported that Map41, one of important recombinant protein of MAP that elicited IFN-γ production in PBMC from cattle infected with MAP [59]. In the study of Reiko and colleagues it was observed that there is an increased production of IL-10 in response to Map41 from experimentally infected calves as early as 2–4 weeks than that of IFN-γ. This characteristic expression of IL-10 in MAP-infected cattle seems to be playing important role in the pathogenesis of paratuberculosis, and could be an important diagnostic indicator [30]. Further studies revealed that the blood cells from calves immunized with MAP produced higher amounts of IL-10 against Map41 stimulation than those calves immunized with various mycobacterium species, in addition, experimentally infected guinea pigs with 14 various mycobacterium species showed high specificity of IL-10 production to MAP after stimulation of splenocytes by Map41 recombinant protein [30]. John et al., [27] also showed that specific MAP recombinant proteins induced an elevated expression of IL-10 by macrophages whereas other proteins are involved in survival of mycobacteria within the macrophages as well as activating the MAPKp38 pathway [27]. M. avium 101 infected C57BLJ6 mice produced peak IL-10 after 2 weeks of infection and remained high throughout the period of infection. Further investigations on the role of IL-10 in the survival of M. avium 101, mice were infected and simultaneously treated with anti-IL-10 antibodies resulted in 2 to 3 log unit less bacterial load in the spleen and liver than the control mice [60]. Another study reported that BALB/c mice were more susceptible to M. avium infection while the reduction of IL-10 activity in these animals through anti-IL-10R mAb, or by using IL-10 knockout mice reduced the susceptibility to M. avium infection. Moreover, the inhibition of IL-10 also increased the efficacy of anti-mycobacterial therapy in infected BALB/c mice [61]. Similarly, when the level of IL-10 was increased by using recombinant IL-10 or IL-10 transgenic mice, animals were more prone to disease [62, 63]. These findings regarding the over production of IL-10 in MAP infected animals as well as MAP or Map recombinant proteins stimulating macrophages suggest the importance of IL-10 in the progression of paratuberculosis.

### The role of IL-10 mediated inhibition of proinflammatory cytokines in paratuberculosis

During mycobacterial infections, cytokine networks triggered by macrophages generally determine the outcome of an infection [64]. IL-10 also inhibits the antimicrobial activity of macrophages by down regulating the expression of IL-12 and TNF-α [34, 65]. Previous studies shown that IL-10 act as a possible mediator of immunosuppressants associated with MAP infection [35, 52]. Investigations on bovine monocytes treated with anti-IL-10 antibodies before challenge with MAP showed an over expression of IL-12, TNF-α and also promoted the bactericidal activity of these cells [65]. Earlier studies reported that IFN-γ and TNF-α plays a key role in the control of intracellular Mtb [64, 66]. IL-12 is one of the important pro-inflammatory cytokine produced primarily by antigen-presenting cells (APCs), including monocytes/macrophages and dendritic cells [67]. IL-12 is composed of p35 (encoded by *Il12a*) and p40 (encoded by *Il12b*) chains, and it principally activates natural killer (NK) cells and induce the differentiation of naive CD4+ T lymphocytes to become IFN-γ producing Th1 effecter cells [67]. IFN-γ, acts on APCs to augment IL-12 secretion in a positive feedback loop [68, 69]. On the other hand, the most important and well studied negative regulator of TLR-induced IL-12 production is IL-10 [70]. IL-10 suppresses the production of IL-12 at transcriptional level by down regulating both *IL12a* and *Il12b* genes [71]. IL-10 overexpressed transgenic mice challenged with Mtb via respiratory route shown a highly significant increase in bacterial number in the lungs, while decreased mRNA production of TNF-α and IL-12p40, and a marked decrease in the secretion of Ag-specific IFN-γ was observed [72].

IL-10 is induced in many situations together with pro-inflammatory cytokines, although the pathways that induce IL-10 expression more likely negatively regulate the expression of these pro-inflammatory cytokines [33]. Dendritic cells (DC) and macrophages produce TNF-α as a primary response of infection and tissue damage, plays an important role in activation and recruitment of leukocytes [73], and has been demonstrated to be involved in the host defence against Mtb [74]. IL-10 requires Stat3, Jak1, and two distinct regions of the IL-10 receptor intracellular domain to inhibit TNF-α production in lipopolysaccharide stimulated macrophages. Macrophages deficient in Stat3 or Jak1 are unable to inhibit lipopolysaccharide induced TNF-α production following treatment with murine IL-10 [75]. IFN-γ is a key molecule that helps in the activation of macrophages with the production of reactive oxygen and nitrogen species, activation of T cells and maturation of DCs [76]. IFN-γ is the early marker of cell-mediated immune response and essential for effective clearance of the mycobacteria, but with the progression of disease, an immunosuppressive cytokine IL-10 becomes predominant [50, 51]. An enhanced host generated IFN-γ and IL-17A were observed in CBA/J mice during BCG vaccination resulted in additional protection against Mtb by inhibition of IL-10 activity through anti-IL-10R mAb treatment [77]. In the clinical stages of paratuberculosis, it was observed that IL-10 suppressed the production of IFN-γ [38]. Neutralization of IL-10 in vitro resulted in increased IL-12, TNF-α, and IFN-γ mRNA expression by bovine monocytes exposed to MAP or purified protein derivative (Johnin) [50]. Mice deficient in IL-10 eliminated M.bovis BCG faster than wild-type mice, while macrophages in the granulomas expressed high level of MHC class II molecule, TNF-α, acid phosphatase and inducible nitric oxide sythase (iNOS) [78]. The serine/threonine protein kinase C (PKC) theta plays an important role in host defence against *Salmonella typhimurium*, as resulted in high number of bacteria in spleen and liver of PKC theta−/− mice. Furthermore PKC theta-deficient macrophage expressed significantly higher levels of IL-10 in in-vivo and in-vitro when challenged with S.typhimurium or LPS/IFN-γ while neutralization of IL-10 improved the control of S.typhimurium infection in PKC theta deficient macrophages [79]. It has been shown that the exogenous treatment of IL-10 inhibit macrophage function like the release of NO and TNF-α, IL-6, and GM-CSF secretion, induced by LPS [80]. Taken together IL-10 enhances the growth and persistent survival of MAP in phagocytic cells by inhibiting the secretion of pro-inflammatory cytokines.

### The role of IL-10 in the shift of immune responses from Th1 to Th2 dominated immune responses in paratuberculosis infection

**Fig. 1:**
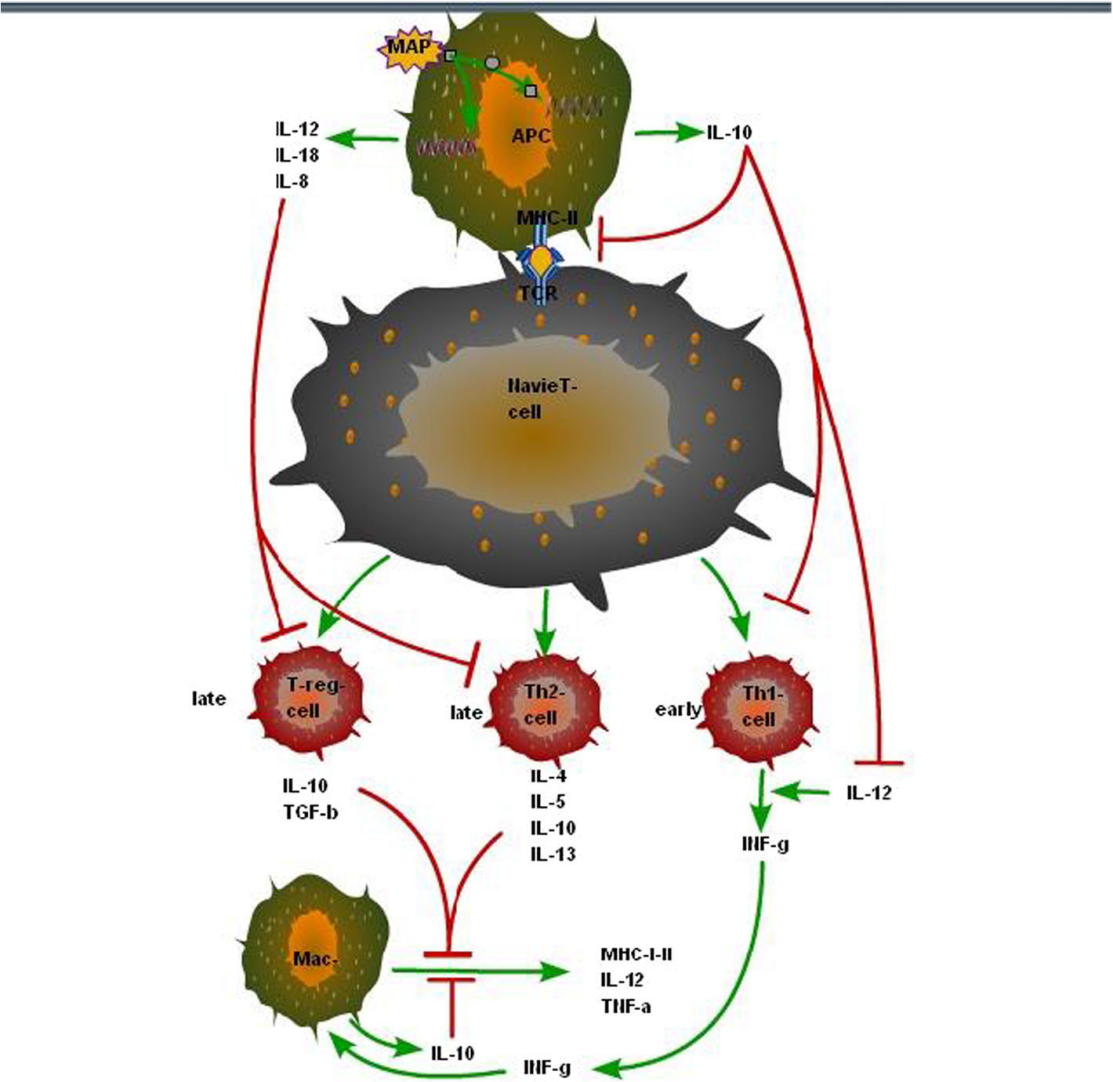
A classical switch between Th-1/Th-2 immune response in paratuberculosis: Navi Th cell differentiate into subpopulation of effectors Th cells when bind to antigen presented by antigen presenting cell (APC). In MAP infection the early differentiated effectors T helper1 (Th1) cell promote protective cell mediate immune response. Th1 cell secrets cytokines such as interferon-gamma (INF-γ) and Interleukin-12(IL-12) and promote the activation of macrophages. In the later stages of infection T helper 2 (Th2) and regulatory T cell are dominant. Th2 and T-reg cell secret anti-inflammatory cytokines such as IL-4, IL-5, IL-10, IL-13 and TGF-β. These anti-inflammatory cytokines block the activation of macrophages and also down-regulate the cytokines secreted by Th1 cell and prevent cell mediated immune response while promote humoral response mediated by Th2 cell

The progression of paratuberculosis from subclinical to clinical form is mainly dependent on a gradual shift in immune response from a cell-mediated Th1 type response to a humoral Th2 response [16]. The naive helper T cells mainly differentiate into either Th1 cells or Th2 cells effecter cells after activation. The Th1 cells secrete IFN-γ and TNF-α and activate macrophages to kill microbes located within the phagosomes. On the other hand Th2 cells secrete interleukins 4, 5, 10, and 13 (IL-4, IL-5, IL-10, and IL-13) and will mainly defend the animal against extracellular pathogens (Fig. 1). Initially, Th2 cells were the main populations of T cells that produce IL-10 in a sustained manner [81, 82]. One theory suggests that the differentiation of naive T cell into subpopulation of Th1/Th2 cells based on the host immune response mediated by polarization of APCs (Fig. 1) after initial contact with an antigen [83]. The early stage of paratuberculosis is controlled by a cell-mediated Th1 response activated by infected macrophages that express IFN-γ, IL-12 and TNF-α [84]. IFN-γ and TNF-α are important cytokines of the Th1 response, enhancing bactericidal activity of macrophages with the production of reactive oxygen and nitrogen species, activation of T cells and maturation of DCs [76, 85]. Another important role for IFN-γ is the induction of IL-12 which further directs CD4 cells to the Th1 subset. Studies have demonstrated that CD4 cells are also the primary source of IFN-γ in MAP infection [86]. In response to mycobacterial infection, different cytokines like IL-12, IL-17 and IL-23 contribute in the development of Th1 cells [87]. During MAP infection, Th1-type response usually occurs early in the infection cycle with an increase production of IFN-γ [88]. De Silva and colleagues reported that IL-10 mediated Th2 response becomes dominant when the disease is in progress, with a decline in Th1 response. Similarly Begg and colleagues reported that during clinical stage of the disease Th2 type immune responses are more dominant than Th1 type. Further studies suggest that there is a classical switch between Th-1/Th-2 immune response in ruminants infected with MAP [17]. During Mtb infection various type of T helper cells (such as Th1, Th2, Th17, and regulatory T cells) are present but Th1 type of cells are more commonly involved in impaired growth and dispersion of Mtb [89]. In sheep MAP infection, paucibacillary lesions have been correlated with a stronger cell-mediated Th1 response than those with multibacillary lesions with a high level of IL-10 expression [88, 90]. A similar trend of higher levels of Th2 type cytokines IL-4, IL-10, IL-2 were observed in cattle with multibacillary lesions [91]. Recent studies also demonstrated that high expressions of IL-10 and TGF-β in the advanced stage of MAP infection with a diffuse granulomatous lesions, suggest the predominance of Th2 response, which shows a switch of Th1 to Th2 response. In addition, an increased expression of IL-10 and TGF-β could have limited Th1 cytokines and cellular infiltration resulting in less tissue pathology but persistence of MAP infection in bullocks [58].

The role of IL-10 predicted with the cytokine signaling model is similar to the cell model where IL-10 can enhance the Th2 response and suppress the Th1 response which promotes the progression of disease [92]. IL-10 is implicated at many levels of tuberculosis disease, such as directing Th2-type cell expansion in the lung [92, 93]. Several studies shown that some molecular mechanisms exist that cause a switch from IFN-γ production to IL-10 by Th1 cells. It is predicted that IL-4 favor the expression of IL-10, also reported by Gesham et al. [92] that IL-4 inhibit Th1 (IFN-γ) response. In Mtb, IL-10 also block the migration of T cell to the site of infection most commonly impairing the recruitment of Th1 cells to the lungs of infected mice [94]. Hence the control of Mtb is negatively regulated by IL-10 and inhibit the progression of early innate to late adoptive Th1 response [94]. Similarly, another intracellular pathogen *Ehrlichia muris* induced early IL-10 production which impaired bacterial clearance in wild-type mice and deteriorate IFN-γ production while in IL-10 deficient mice IFN-γ production by CD4 T cell was restored and induced a protective immune response [95]. The lack of IL-10 production by T cells resulted in an increased ability of mice to control intracellular *Brucella abortus* infection and elevated the production of pro-inflammatory cytokines. Moreover it is suggested that early IL-10 production by CD25 + CD4+ T cells modulates macrophage activation that is beneficial for bacterial survival and persistent infection [96]. In the clinical form of paratuberculosis, it is observed that Th2 type response dominate with an increased level of cytokines such as IL-4, IL-5 and IL-10 [84, 97]. The dynamics of IL-10, TGF-β and IFN-γ gene expression in the ileum, ileocecal junction, ileocecal lymph node, and mesenteric lymph nodes of healthy cows as well as those in subclinical and clinical stages of infection. IL-10 and TGF-β mRNA levels were significantly higher in all tissues of cows with clinical disease. In contrast, IFN-γ gene expression was significantly higher only in animals with subclinical infection [52]. These reports suggest that IL-10 production during paratuberculosis gradually shift protective Th1 cell mediated immune response to humoral response mediated by Th2 cells.

### The role of IL-10 mediated inhibition of phagosome maturation in paratuberculosis

Phagocytosis is an essential process by which macrophages engulf invading pathogens, apoptotic cells and other foreign particles. Phagocytosis triggers the activation of multiple transmembrane signaling pathways that lead to the formation of a sealed intracellular compartment called phagosome [98]. Pathogenic species of mycobacteria have evolved mechanisms that enhance their survival ability within macrophages by inhibiting acidification and maturation of phagosomal compartment which is necessary for the progression of a phagosome into a functional phagolysosome [99, 100]. MAP adopts multiple strategies for their intracellular survival; one of them involves the inhibition of phagosomal acidification and maturation. MAP blocks the phagosome maturation into phagolysosome by interfering the localization of lysosomal markers and prevents the pH to go below 6.3 in J774 macrophages [101]. Previous studies reported that MAP enhances the phosphorylation of MAPK-p38 in bovine monocytes, resulted in the inhibition of phagosomal acidification and killing of MAP to over-express IL-10 [24]. While the inhibition of p38 phosphorylation by the application of pharmacologically active substances enhance the bactericidal activity of bovine monocytes against MAP organism and increase the acidification of phagosome [21, 24, 35]. IL-10 negatively regulates the maturation of phagosome in Mtb [46]. Man-LAM from MAP interacts with PRRs of macrophages promote IL-10 production and further inhibit phagosomal acidification [26]. Other studies showed that monocytes treated with p38 inhibitor before challenging with MAP and Mtb promote phagosomal maturation and also inhibit IL-10 production [41, 46]. In addition, macrophages treated with cytokines lead to an increase in the acidification and maturation of phagosomal compartments containing MAP [102]. Furthermore, phagosome maturation was enhanced by blocking STAT3 activity in Mtb-infected macrophages [46]. The acidification of phagosomal compartment in macrophages infected by pathogenic mycobacteria is inhibited through the elimination of phagosomal proton pump, the Vacuolar Hþ ATPase (V-ATPase), from their phagosomal membranes [103]. Phagosomal body having MAP or Mtb are deficient in V-ATPase [99]. It is clear from the above findings that p38 phoshorylation promote IL-10 production and further increase MAP resistance to defence mechanisms of the host by blocking the acidification and maturation of phagosomal compartments.

### Role of IL-10 mediated apoptosis in paratuberculosis

The process of programme cell death (apoptosis) is also an essential mechanism of the host for the control of intracellular infections [103]. However, some pathogens may prevent host cell apoptosis, circumventing efferocytosis, and ensuring limited immune system detection [104]. MAP keeps the host cell alive by preventing apoptotic suicide of macrophages. Macrophages infected with mycobacteria tend to activate apoptotic pathways, resulting in cell death [105]. However, pathogenic strains of mycobacteria induce less apoptosis as compared to low pathogenic strains. For example, bovine monocyte-derived macrophages (BMDM) have a greater percentage of apoptotic cells when incubated with the less pathogenic organism MAA in comparison to bovine pathogen MAP [106]. Pathogenic mycobacteria also limit apoptosis by inhibiting TNF-α expression and releasing soluble TNF-receptor-2 (TNFR2) that neutralize TNF-α activity [105]. This is accomplished primarily through over expression of IL-10 [35, 107]. IL-10 not only suppresses TNF-α synthesis but also enhance soluble TNFR2 [105]. High concentration of IL-10 also impairs apoptosis induced by Mtb in alveolar macrophages [108]. IL-10 significantly up-regulates the expression of suppressor of cytokine signaling-3 (SOCS3), and modulates oxidized low-density lipoprotein (oxLDL) to inhibit apoptosis in endothelial cells [109]. It has been reported that IL-10 inhibits the production of inflammatory cytokines by macrophages and protect cell from apoptosis [110]. In addition, IL-10 induces its anti-apoptotic properties via overexpression of SOCS3 that inhibit inflammatory cytokine involved in the progression of apoptosis. This phenomenon was also reported in a recent study showing that SOCS3 and SOCS1 suppressed cytokine-induced apoptosis [111]. IL-10 also prevents apoptotic cell death of infected macrophages by inhibiting multiple components that positively regulate apoptosis phenomena and promote the survival of MAP.

## IL-10 regulating signaling pathways

Phagocytic cells have various pathogen recognition receptors (PRRs) for initiating signaling cascades. Among different PRRs TLRs are important pattern recognition receptors of phagocytic cells, signaling pathways followed by TLR2 and TLR4 are potent inducers of IL-10 [112]. It has been suggested that TLR2 agonists are specialized in inducing IL-10 expression by APCs [112, 113]. Several studies indicate that pathogenic mycobacteria signal through TLR2 is crucial for the induction of IL-10, suppress macrophage antimicrobial responses and antigen presentation [114]. TLR2/4 is major signaling receptors of phagocytic cells activate MAPK pathway and may activate the NF-kB pathway after ligation with Man-LAM and 19 kDa of MAP [25]. The common signaling pathways that induce the regulation of IL-10 in phagocytic cells are MAPK, NF-kB, and STAT [25, 43].

### MAPK pathway

Mitogen-activated protein kinase (MAPK) is a signal transduction pathway belongs to a large family of serine/threonine kinases, which constitute major inflammatory signaling pathways from the cell surface to the nucleus [115]. There are three well-characterized subfamilies of MAPKs: the extracellular signal-regulated kinases (ERK), the c-Jun NH2-terminal kinases (JNK), and the p38 family of kinases (p38 MAPKs) [116, 117]. Both ERK and p38 are crucial for the Mtb-induced production of TNF-α, while p38 is more essential for the secretion of IL-10 in macrophage [40]. MAP induced IL-10 expression by bovine monocytes is mediated through activation of MAPK-p38, but not MAPK-ERK, or MAPK-JNK [24, 41, 105]. Blocking the MAPK-p38 pathway inhibits the capacity of MAP to induce IL-10 expression, suppress IL-12, block phagosome acidification, and inhibit organism killing [41]. MAPK-p38 kinase exists as four isoforms, alpha, beta, gamma and delta [118]. In case of p38 alpha, it is the sites Thr180/Tyr182 that become dual phosphorylated, signaling activation within the cell. Following TLR ligation, signaling cascades are activated through Toll/IL-1 receptor (TIR)-domain-containing adaptor molecules, such as myeloid differentiation primary response protein 88 (MYD88) which consists of a C-terminal TIR and an N-terminal death domain (DD). The later interacts with the DD of a serin/threonin protein kinase, IL-1 receptor-associated kinases (IRAK). Binding to MyD88 enables IRAK4 to phosphorylate the subsequently recruited IRAK1, thereby inducing the activation of its kinase activity. Autophosphorylated IRAK1 subsequently dissociates from the receptor complex and associates with TIR-domain containing adaptor protein inducing IFN-β 6 (TRAF6) [119]. The IRAK1-TRAF6 complex interacts at the plasma membrane with another preformed complex consisting of TGF-β-activated protein kinase 1 (TAK1) TAK binding protein1 (TAB1) and TAB2 or TAB3. This interaction induces phosphorylation of TAB2/TAB3 and TAK1, leading to their translocation to the cytoplasm together with TRAF6 and TAK1, whereas IRAK1 is degraded at the plasma membrane. In the cytoplasm, TAK1 is then activated, resulting in the activation of the MAPK-p38, ERK and JNK. TAK1 activation also, resulting in the activation of IKKs (IkB kinases), which then phosphorylate the IκBs (inhibitor of NF-κB). This phosphorylation allows ubiquitylation and subsequent degradation of IκB, thereby releasing NF-κB. NF-κB is consequently free to translocate into the nucleus and induce the expression of its target genes [120]. These down-signaling cascade leading to activation and nuclear translocation of transcription factors such as specific protein 1(Sp1) [121], activator protein (AP), CCAAT/enhancer binding protein(C/EBP) β and δ [122], c-musculoaponeurotic fibrosarcoma factor (c-Maf) [123], nuclear factor κ-B (NFκB) [124], and phosphorylated cyclic AMP element binding protein (CREB) [125] playing critical role in the expression of IL-10. Although, MAPK-p38 pathway is an antimicrobial suppressive response within macrophages, as a result the activation of this pathway could enable intracellular survival of MAP and also induce the production of the anti-inflammatory cytokine such as IL-10 [41, 69].

### NF-κB pathway

Several studies suggested that NF-κB plays a role in regulating IL-10 production. There are five rel proteins in NF-kB: RelA (p65), RelB and cRel (which contain transactivation domains) and p50 and p52 {which are expressed as the precursor proteins p105 (NF-κB1) and p100 (NF-κB2)} [126]. Gene knockout studies have also shown that NF-kB proteins may have both pro and anti-inflammatory roles. Homodimers of the p50 subunit of NF-kB, which lack transactivation domains, have been shown to repress expression of NF-kB target genes and inhibit inflammation [127]. Further studies have shown that administration of IL-10 fusion protein inhibited IL-12p40 production dependent on p50/p105 expression in macrophages [128]. These studies suggest that NF-kB may have anti-inflammatory roles by directly inhibiting the expression of pro-inflammatory genes and by manipulating the expression or activity of anti-inflammatory cytokines such as IL-10 [127]. The transcription of IL-10 is also promoted by one of NF-kB family member that is NF-kB1 (p50), where p50 can homodimerize and form a complex with the transcriptional co-activator CREB-binding protein to activate transcription, while other Rel family members appear to play a negligible role in IL-10 transcription [129]. NF-κB1-deficient macrophages have lower levels of IL-10 expression than control cells following TLR activation [112].

### STAT pathway

Signal transducer and activators of transcription (STATs) are a family of transcription factors that play crucial roles in regulating a number of diverse biological functions including cell proliferation, differentiation, apoptosis, inflammatory response, immunity, and angiogenesis [130]. There are seven STAT proteins (STATs 1, 2, 3, 4, 5a, 5b, and 6), which are activated by the signals from cytokine and growth factor receptors in the plasma membrane and regulate gene transcription [131]. A unique feature of STAT proteins includes signal transduction through the cytoplasm and functioning as transcription factors in the nucleus [132]. STAT proteins are activated by phosphorylation of specific tyrosine residues, following which they form stable homodimers or heterodimers with other STAT proteins through reciprocal phosphotyrosine-SRC homology 2 (SH2) domain interactions [133]. STAT dimers then translocate to nucleus where they regulate the transcription of a set of specific genes. STAT3 is unique of all STAT family protein, play important role as a signaling mediator of IL-6 and IL-10 family members and other cytokines such as leptin and G-CSF [133]. IL-10 also induces IL-10 in monocyte-derived macrophages in an autocrine manner via activation of STAT3 factor [45]. IL-10 bind to IL-10R1/IL-10R2 receptor complex that will lead the phosphorylation of cytoplasmic protein Janus kinase (JAK) 1(associated with the IL-10 receptor α chain) and phosphorylation of tyrosine kinase (Tyk) 2 (associated with the IL-10 receptor β chain) leads to the phosphorylation and activation of transcription factor, STAT3 [43), resulting in up-regulation of IL-10 responsive genes, such as SOCS3, and downregulation of a number of LPS-inducible genes including pro-inflammatory cytokines in peripheral blood mononuclear cells [134]. STAT3 is critical regulator of IL-10 and have been proposed to transactivate *IL10* gene in macrophage and T cell lines of mouse or human origin [135]. IL-10 mediates its anti-inflammatory effects on macrophages by signaling through STAT3 [43, 45]. STAT3 has been reported to directly activate transcription of IL-10 and TGF-β genes [136]. Moreover, STAT3 has been shown to compete for binding of NF-kB Rel to the IL-12p35 promoter inhibiting its transcription [137]. Genetic and biochemical evidence from both human and mouse systems indicate that STAT3 is required to generate IL-10-induced anti-inflammatory responses [43].

## The role of miRNA in regulation of IL-10 and other signalling pathways in immune cells in paratuberculosis infection

The best possible therapeutic strategy towards achieving the goal of targeting MAPK pathway by itself or its downstream signaling in paratuberculosis can be through the regulation of IL-10 directly or via STAT3 pathway. The miRNAs are small, non-coding RNAs molecules that bind complementary sequences in the 3 untranslated regions (UTRs) of the target genes [138]. In the immune system miRNAs play an important role in the development and differentiation of hematopoietic subset of cells like, DC development [139]. Recent studies found that miRNAs have critical role in both innate and adoptive immune response [140], such as macrophage polarization, T cell and B cell differentiation [141]. The role of miRNAs has been investigated in the regulation of IL-10 and its related pathways in immune modulating cells [142, 143]. Feng et al., [144] demonstrated that miR-466 l up-regulate IL-10 expression in TLR-triggered macrophages both at mRNA and protein levels. Some miRNAs are IL-10–dependent such as miR-187, playing a role in IL-10 mediated suppression of TNF-α, IL-6, and the p40 subunit of IL-12 (IL-12p40) produced by primary human monocytes following activation of TLR4 [145]. On the other hand several studies investigated that miRNAs also negatively regulate the expression of IL-10 and can be used to neutralize the anti-inflammatory effect of IL-10 in different immune suppressive diseases. In addition to miR-155, miR-147 also counter regulates IL-10 subsequently promoting pro-inflammatory functions in TLR-triggered macrophages [146]. The overexpression of miR-98 inhibited TLR4-triggered IL-10 production and promoted COX-2 expression, by targeting the 3’untranslated region of IL-10 transcripts [147]. Similarly, exogenously mimic miR-27a negatively regulates the expression of IL-10 in macrophages stimulated by LPS, while promote pro-inflammatory cytokines [148]. MiR-689, miR-124, and miR-155 were the most strongly associated miRNAs predicted to mediate pro-inflammatory pathways and activation of M1 phenotype of macrophage [149]. In terms of both phenotype and function, macrophages display two well established polarized phenotypes are referred to as the classically activated macrophages (M1) and the alternatively activated macrophages (M2) [150]. M1 phenotype produce large amounts of pro-inflammatory cytokines, express high levels of major histocompatibility complex molecules and are potent killers of pathogens and tumour cells [151], while M2 phenotype, usually activated by the IL-4/IL-13 immune complex, IL-10, or TGF-β, are associated with an immunosuppressive phenotype, an enhanced release of anti-inflammatory cytokines [142]. As reported by Ouiment and colleagues that, miR-33 promote M1 phenotype by up-regulating the expression of M1 markers such as *Il6*, *Nos2*, and *Il1b,* while reduced the expression of the M2 macrophages markers, *Mrc1* (mannose receptor CD206) and *Fizz1/Retnla* [152]. Sisi et al., [143] reported that miR-23a and 27a inhibit the polerization of M2 phenotype while promote M1 phenotype by targeting 3UTR of JAK1/STAT6 and IRF4/PPAR-γ. The cluster of miR-23a/27a/24-2 enhance the production of inflammatory mediators (IL-1b, IL-6, IL-12 and TNF-a) while inhibit anti-inflammatory cytokine such as IL-10 [144]. In addition to that other studies reported that miRNAs, such as miR-155, miR-146a and miR-9 negatively regulated the activation of MAPK-p38 and JNK signaling pathways [153]. The activation of JAK-STAT3 pathway playing important role in the regulation of IL-10 and also inhibit anti-bactericidal activities of phagocytic cells. Recently it has been determined that miRNAs also have role in the regulation of STAT3 expression, such as 23a/27a/24-2 got therapeutic significance by inhibiting JAK1-Stat3 and synergistically promote apoptosis [154]. Recently it is demonstrated that overexpression of miR-124, miR-519a and miR-351 inhibit STAT3 by targeting 3 untranslated region of STAT3 [154, 155]. The miR-204 also inhibits the activation of STAT3 and BCL2 by targeting JAK2 [156].

Apart from miRNAs there are some other compounds that have role in the regulation of IL-10 or IL-10 dependent pathways in several diseases, such as ammonium trichloro (dioxoethylene-O, O’) tellurate (AS101) inhibit IL-10 production by dephosphorylation of Stat3, followed by reduced expression of Bcl-2 [157]. In addition AS101 deactivate Akt that leads to a decrease in pGSK3 expression and reduce IL-10 production [158]. Sinuani and colleagues reported that AS101 also negatively regulate TGF-β in uninephrectomized rats [159]. AS101 also got immunemodulatory properties by inhibiting anti-inflammatory cytokines, IL-10 while promote pro-inflammatory cytokines [160]. On the other hand, Myers et al., found that SD-169, a novel oral inhibitor of MAPK-p38 [161]. Another important compound that inhibits the activation of MAPK/ERK1/2 and IκB kinases is niclosamide, an FDA-approved anti-helminthic compound, also down-regulate Stat3 and Bcl-2 family members such as Mcl-1 [162]. You et al., [163] also investigated the role of niclosamide in the progression of lung cancer by blocking STAT3/Bcl2/Bcl-Xl signaling pathway. These compounds can be used to control the progression of paratuberculosis by targeting IL-10 regulating pathways in mononuclear phagocytic cells.

## Conclusions

*Mycobacterium avium* subspecies *paratuberculosis* (MAP) is the causative agent of paratuberculosis. MAP not only lead to high economic losses in livestock sector but also a potential zoonotic threat by considering a contributing factor in Crohn’s disease of humans. The hallmark of MAP reactivation is the failure of the host immune response to restrain the bacterial growth. The most critical immune evasion mechanism of MAP survival in macrophages is the activation of MAPK-p38 dependent TLR2-MyD88 adopter protein. MAPK-p38 activation inhibits antimicrobicidal activity of macrophages and over expression of IL-10. It is understood that IL-10 plays a critical role in maintaining homeostasis in the body by resolving inflammation during acute infection or any injury, while high level of IL-10 in paratuberculosis promote the survival of MAP. The excessive amount of IL-10 reduces the bactericidal ability of defense cells. IL-10 is used as a weapon by the bacilli to interfere with proper macrophage activation during active infections. The pathways involved in the upregulation of IL-10 such as MAPK can be vital for developing a therapeutic strategy for the control of paratuberculosis. Recent studies highlighted the role of miRNAs in pathogen–host interactions. It has been investigated that certain miRNAs positively regulates the host immune response against Mtb. Similarly, different miRNAs regulates various signaling components of MAPKp-38 pathway as well as anti-inflammatory cytokines. Recent studies on profiling of miRNAs during clinical and subclinical infection of MAP gave a new perspective to MAP control via miRNA techniques. These studies shown that circulating miRNAs in MAP infected animals retain their integrity under longterm suboptimal storage temperatures and are considered as potential biomarkers [164, 165] that can be exploited for devising the future paratuberculosis control strategies. Apart from miRNAs several compounds such as AS101, SD169 and niclosamide can be used to target IL-10 mediated anti-inflammatory activity. The knowledge reviewed about the mechanism of MAP pathogenesis will likely lead to a better understanding of paratuberculosis treatment and control.

## Abbreviations

TGF-β: Transforming growth factor-beta
TRAF6: TIR-domain containing adaptor protein inducing IFNβ 6
AP: Activator protein
APCs: Antigen-presenting cells
AS101: Ammonium trichloro (dioxoethylene-O, O’) tellurate
Bcl2: B-cell lypmphoma2
BMDM: Bovine monocyte-derived macrophages
CD: *Morbus crohn* disease
CREB: Cyclic AMP element binding protein
DD: Death domain
ERK: Extracellular signal-regulated kinases
IFN-γ: Interferon-gamma
IL-10: Interleukin-10
IL-12: Interleukin-12
IRAK: IL-1 receptor-associated kinases
IκBs: Inhibitor of NF-κB
JAK: Janus Kinase
JNK: c-Jun NH2-terminal kinases
LPS: Lypopolysaccharide
MAA: *Mycobacterium avium* subsp *avium*
Man-LAM: Mannosylated lipoarabinomannan
MAP: *Mycobacterium avium* subsp. *Paratuberculosis*
MAPK: Mitogen-activated protein kinase pathway
MDM: Monocytes derived macrophages
miRNAs: Micro-RNAs
Mtb: *Mycobacterium tuberculosis*
MYD88: Myeloid differentiation primary response protein 88
NF-kB: Neuclear factor kappa-B
NK cells: Natural killer cells
NO2: Nitic oxide
oxLDL: oxidized low-density lipoprotein
PAMP: Pathogen associated molecular pattern
PBMC: Peripheral blood mononuclear cells
PRRs: Pathogen recognition receptors
SOCS3: Suppressor of cytokine signaling-3
Sp1: Specific protein 1
STAT3: Signal transducers and activators of transcription 3
TAB1: TAK binding protein1
TAK1: TGF*β*-activated protein kinase 1
Th1: T-helper1
Th2: T helper2
TIR: Toll/IL-1 receptor
TLR2: Toll like resceptor
TNF-α: Tumor necrosis factor alpha
Tyk: Tyrosine kinase 2
UTRs: Untranslated regions
